# Correction: Standardized Extubation and High Flow Nasal Cannula Training Program for Pediatric Critical Care Providers in Lima, Peru

**DOI:** 10.15766/mep_2374-8265.11007

**Published:** 2020-09-11

## Correction

In the publication “Standardized Extubation and High Flow Nasal Cannula Training Program for Pediatric Critical Care Providers in Lima, Peru,” three author names were incorrectly formatted in the citation field. The correct citation is shown below:

Ellington LE, Becerra R, Tantaleán da Fieno J, Malma G, Nielsen KR. Standardized extubation and high flow nasal cannula training program for pediatric critical care providers in Lima, Peru. *MedEdPORTAL*. 2020;16:10937. https://doi.org/10.15766/mep_2374-8265.10937

## References

[R1] Ellington LE, Velásquez RB, da Fieno JT, Arrescurrenaga GM, Nielsen KR Standardized extubation and high flow nasal cannula training program for pediatric critical care providers in Lima, Peru. MedEdPORTAL. 2020;16:10937 10.15766/mep_2374-8265.10937 32782926 PMC7412765

